# HIV and immunotherapy: will CAR-T cell therapy cure HIV?

**DOI:** 10.1097/JS9.0000000000000556

**Published:** 2023-06-14

**Authors:** Vishnu P. Veeraraghavan, Jyotsna Needamangalam Balaji, Sreenidhi Prakash, Lavina Prashar, Ullas Mony, Krishna M. Surapaneni

**Affiliations:** a Centre of Molecular Medicine and Diagnostics (COMManD), Saveetha Dental College and Hospitals, Saveetha Institute of Medical and Technical Sciences, Saveetha University; b Panimalar Medical College Hospital & Research Institute; c Departments of Biochemistry, Medical Education, Molecular Virology, Research, Clinical Skills & Simulation, Panimalar Medical College Hospital & Research Institute, Varadharajapuram, Chennai, Tamil Nadu, India; d Department of Physiological Sciences, School of Medicine, Unza Ridgeway Campus, University of Zambia, Lusaka, Zambia

*Dear Editor,*

As the global burden of AIDS and associated complications continue to rise, so are the advancements made in the therapeutic approaches towards combating the HIV^1^. According to UNAIDS - 2021 statistical report, around 38.4 million people worldwide live with HIV infection with nearly 650 000 deaths due to AIDS-related illness^2^. To reduce the morbidity and mortality due to HIV, newer and more effective immunotherapeutic techniques are invented with one of the recent approaches being Chimeric Antigen Receptor (CAR) - T cell therapy^3^.

CAR-T cell therapy has demonstrated promising results in haematological malignancies and is also emerging as the standard treatment for different types of leukaemia, nonhematopoietic malignancies, and other infectious diseases. Recently, owing to its well-established outcomes in the treatment of cancers, rigorous efforts have been made to reciprocate the same in HIV cure^4^. The unique feature of this targeted therapy is that it multiplies and engrafts to the extent necessary for a sustained therapeutic effect thereby boosting the immunogenic memory and monitoring of viral growth. Unlike other therapeutic objectives, this method is predicted to be most advantageous in terms of completely eliminating cells infected with HIV and preventing viral resurge^5^.

The mechanism by which CAR-T cell therapy works sounds promising. Cytotoxic T Lymphocytes are key components in regulating the breakdown of infected cells along with the major histocompatibility complex class I (MHC-I) molecules. But HIV has the potential to suppress the surface expression of MHC-I in infected lymphocytes to evade this defence mechanism thereby leading to massive immunodeficiency and disease progression, making conventional therapy incapable of providing a complete cure to this condition. This is where the distinctiveness of CAR-T cells is seen. As the CAR recognises antigens directly without the MHC-I limitation, these cells are capable of circumventing the viral escape mechanism with a prolonged duration of action making it a reliable therapeutic approach in the complete elimination of viral load^5,6^.

However, despite this approach demonstrating positive outcomes *in vitro*, the desired therapeutic results in actual clinical practice have not been substantiated. A clinical trial using CAR-T for the treatment of HIV started in UC Davis Medical Centre, USA to identify a potemtial cure for HIV. Although this seems a promising approach, there are several challenges in the way ahead. CAR-T cell expansion, neurotoxicity, off-target effects, severe cytokine release syndrome, cost and scalability remain potential drawbacks of this technique^6^. Combination strategies, including employing CAR-T cell treatment together with other immunotherapies or latency-reversing medications, may offer favourable solutions but more evidence-based results are needed in this regard. Thus, owing to its remarkable utility in the treatment of malignancies, deeper research and collaboration to prove the efficacy of CAR-T cell therapy in achieving a functional cure for HIV infection is certainly worth time and effort.

## Ethical approval

Not applicable.

## Source of funding

Not funded by any funding agency.

## Author contribution

V.P.V.: conceptualization, data curation, writing-original draft preparation; J.N.B.: data curation, writing-original draft preparation; S.P.: data curation, writing-original draft preparation; L.P.: data curation, writing-original draft preparation; U.M.: data curation, writing-original draft preparation; K.M.S.: conceptualization, study design, data curation, writing-original draft preparation, reviewing and editing, visualisation and supervision.

## Conflicts of interest disclosure

There are no conflicts of interest.

## Guarantor

Dr Surapaneni Krishna Mohan.

## Data availability statement

This correspondence is based exclusively on resources that are publicly available on the internet and duly cited in the ‘References’ section.

No primary data was generated and reported in this manuscript. Therefore, data has not become available to any academic repository.
